# Incidental Findings on Cone Beam Computed Tomography Images

**DOI:** 10.1155/2012/871532

**Published:** 2012-12-10

**Authors:** Veeratrishul Allareddy, Steven D. Vincent, John W. Hellstein, Fang Qian, Wendy R. K. Smoker, Axel Ruprecht

**Affiliations:** ^1^Department of Oral Pathology, Radiology, and Medicine, The University of Iowa, 801 Newton Road, Iowa City, IA 52242, USA; ^2^Department of Preventive and Community Dentistry, 801 Newton Road, Iowa City, IA 52242, USA; ^3^Department of Radiology, The University of Iowa, 200 Hawkins Drive, Iowa City, IA 52242, USA

## Abstract

*Background*. Cone beam computed tomography (CBCT) has gained widespread acceptance in dentistry for a variety of applications. Most dentists who are not radiologists/trained in radiology are generally not familiar with interpretation of anatomical structures and/or pathosis outside their area of primary interest, as often this was not within the scope of their training. *Objectives*. To assess that the number of incidental findings on a CBCT scan is high both within and outside of the primary area of interest, thereby emphasizing the importance of interpretation of all areas visualized on the scan. *Materials and Methods*. An oral and maxillofacial radiologist reviewed 1000 CBCT scans (382 males and 618 females) for findings both in- and outside the area of interest. *Results*. Of the 1000 subjects that were reviewed, 943 scans showed findings in the primary regions of interest and/or outside the regions of interest, and 76 different conditions were visualized in these scans both in and outside the areas of interest. *Conclusion*. From the wide scope of findings noted on these scans, it can be concluded that it is essential that a person trained in advanced interpretation techniques in radiology interprets cone beam computed tomography scans.

## 1. Introduction

The development of panoramic radiography in the 1950s and its commercial introduction in 1965 led to the widespread adoption and use of the technology. Although widely used, these images have the same inherent limitations as other 2D projections, namely, magnification and minification of structures, superimposition of anatomical and/or pathological entities, and misrepresentation of structures. However panoramic radiography is efficient at providing an overview of oral and maxillofacial hard tissues, including teeth, and may reveal associated pathoses of the jaws. To overcome some of the above limitations cone beam computed tomography (CBCT) for the jaws was developed in the 1990s and is gaining widespread acceptance in dentistry, especially in the last 5 years. 

CBCT was initially developed for angiography and is also popularly used for radiotherapy guidance and mammography [1] as an alternative to conventional fan beam helical computed tomography (CT) machines to provide more rapid acquisition of a dataset of the entire field of view. This rapid acquisition has enabled less movement in patients during the process of acquisition of the images. In addition, the radiation dose to the patient is lower than that of conventional CT machines and CBCT machines are markedly less expensive than conventional CT machines.

However, there are disadvantages. The field of view is usually smaller than that of standard CT and there is a lack of differentiation among various soft tissues. 

CBCT scanners were first introduced in 1997 in Italy [2]. Cone beam computed tomography scanners have been commercially available since 2001 in the United States [3]. 

CBCT is used in dentistry for a variety of applications such as

- evaluation of pathosis in the jaws,
- evaluation of bone for implants,
- orthodontic assessments,
- tmj assessments, and
- endodontic assessments.

This imaging modality is mainly for the osseous and dental components of the maxillofacial complex. 

Most dentists who are not radiologists are not familiar with interpretation of anatomical structures and/or pathosis outside the area of primary interest. This study was undertaken to determine the prevalence and nature of unusual findings in and around the maxillofacial complex encountered in CBCT studies.

## 2. Aim

To assess whether the number of incidental findings on a CBCT scan is high inside and outside the primary areas of interest as well, thereby emphasizing the importance of interpretation of all areas visualized on the scan.

## 3. Materials and Methods

### 3.1. Source of the Scans

The CBCT scans (Table 2) used in the study were acquired on an i-CAT machine (Imaging Sciences International, Hatfield, PA). To assess the feasibility of this study, a pilot survey was carried out on a sequential series of 100 subjects scanned at a private imaging company. Institutional Review Board approval to use the information on the scans for the study was obtained at The University of Iowa. Once the data obtained from the initial 100 subjects showed the feasibility and importance of a comprehensive study, a series of 1000 subjects who were imaged at the same company were analyzed comprehensively. Consent was obtained from all the subjects as well as the private imaging company to use and share the information from the scan for purposes of education, including for teaching and research.

### 3.2. Field of View of the Scans

All the scans used for this study were acquired at a field of view of 13 cm and a 0.3 mm thickness. 

### 3.3. Time of Exposure to Acquire the Scan

The time of exposure to acquire the scan was uniform at 8.5 seconds for 843 subjects and 20 seconds for 157 subjects after acquiring the scout image to assess and adjusted for proper positioning of the subject to include the region of interest in the scan. The field of view and thickness of the slices remained the same in both sets of subjects despite the change in scan time from 20 seconds to 8.5 seconds. The decrease in time of acquisition was due to the increased image acquisition speed of a new sensor. The field of view and the slice thickness remained the same for all the subjects.

### 3.4. Age and Sex Distribution of Subjects

The age range of subjects is 5 years and 11 to 87 years. See Table 1 for the entire list of ages of the subjects.

### 3.5. Reasons for the Scan

The subjects scanned presented for a variety of reasons given in Table 1

### 3.6. Interpretation and Review of the Scans

An oral and maxillofacial radiologist (VA), using the proprietary i-CAT viewer software version 3.034, reviewed the images. If there was any doubt on any of the findings, other authors were consulted for their opinion.

## 4. Results

Table 3 represents the pathologic entities and/or anatomic variants found inside and out of the primary region of interest. For convenience they have been grouped into different headings in the table. 

**Figure 1:**
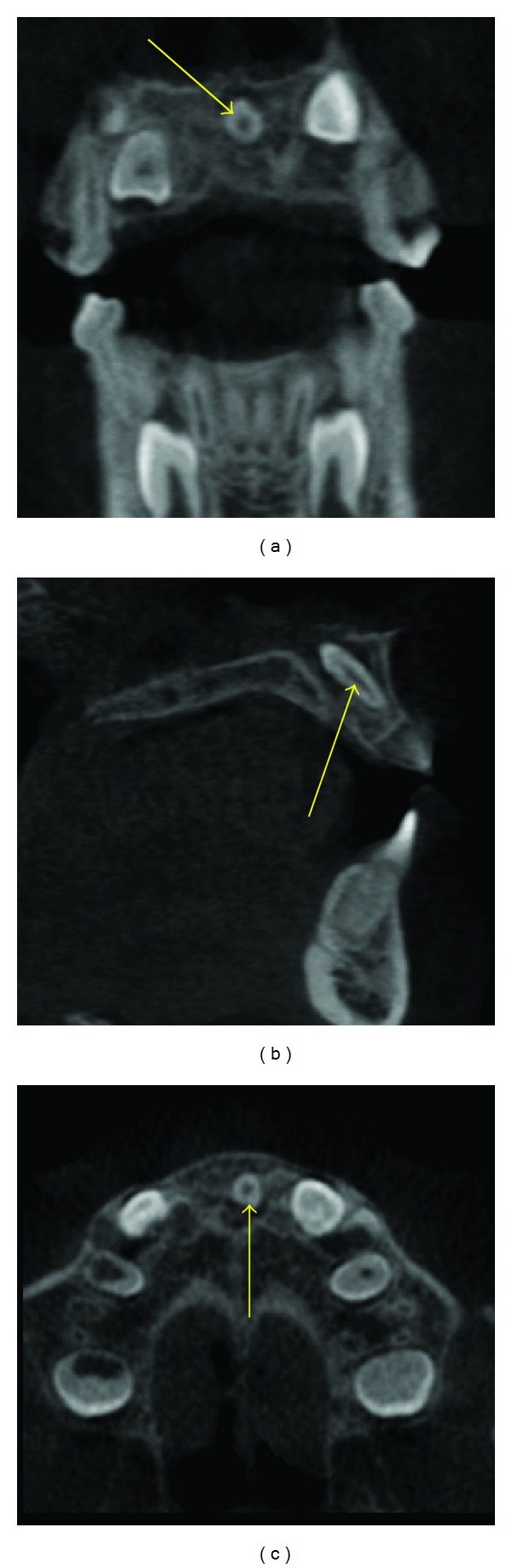
An impacted mesiodens (arrows) angulated obliquely such that the crown is towards the floor of the nasal cavity and the root between the roots of the central incisors of the maxilla is seen in this MPR image.

**Figure 2:**
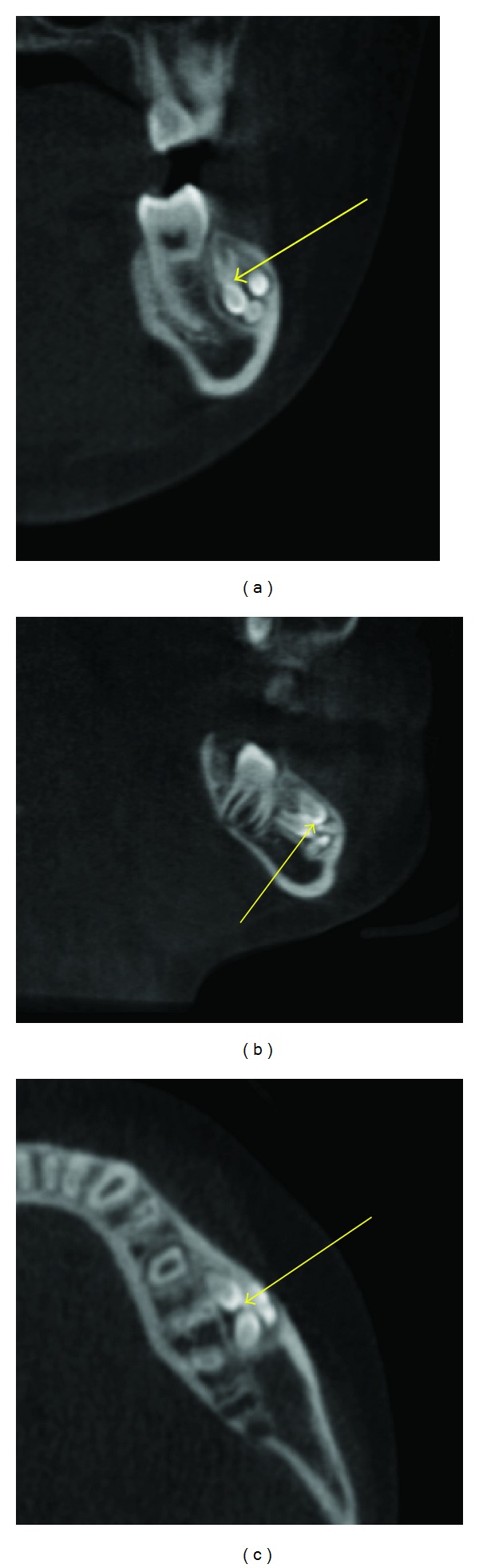
A Compound odontoma (arrows) made of multiple small tooth like entities seen in the facial aspect of the mandibular left second premolar and molar regions as seen in the mandible on these MPR images.

Eighty nine subjects had variations in size, shape, and number of teeth. Amongst these subjects the most common finding was missing teeth (38 subjects). The other more common finding was the presence of supernumerary teeth (31 subjects) (Figure 1). Transposition (5 subjects), compound odontomas (Figure 2) (4 subjects), microdontia (4 subjects) were the other relatively common findings. It was interesting to note that rare entities like dentin dysplasia (2 subjects), dentinogenesis imperfecta (1 subject), amelogenesis imperfecta (1 subject) were encountered amongst the scans reviewed. Taurodontism (1 subject), gemination (1 subject), and macrodontia (1 subject) were the other conditions encountered amongst these patients as far as variations in shape and size of teeth were concerned.

**Figure 3:**
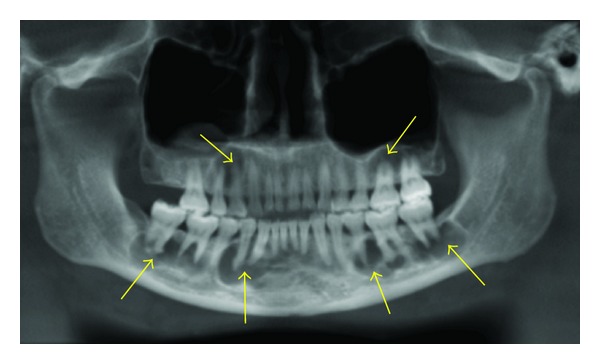
Florid cemento-osseous dysplasia seen as mixed predominantly radiolucent lesions seen in the periapical regions of most of the teeth in the maxillae and the mandible on this panoramic reconstruction image.

**Figure 4:**
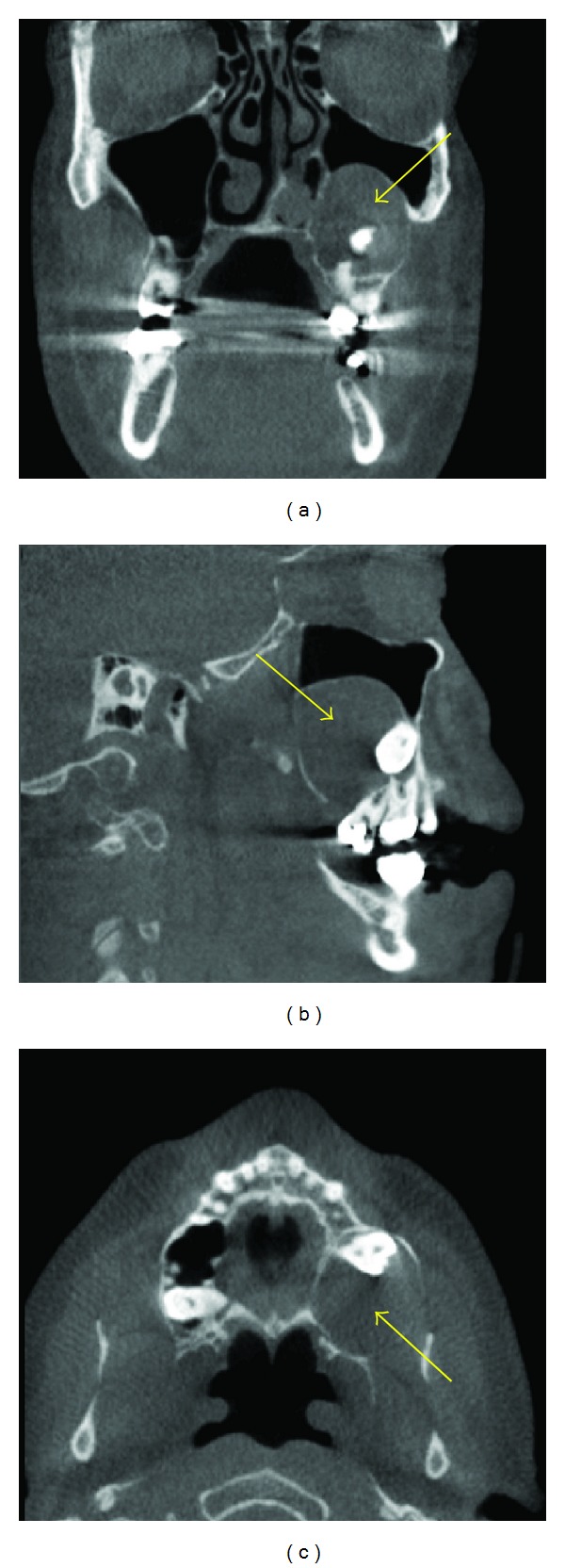
A dentigerous cyst (arrows) seen as well defined radiolucent entity around the crown of the impacted and displaced maxillary left third molar and is encroaching into the left maxillary sinus in these MPR images.

**Figure 5:**
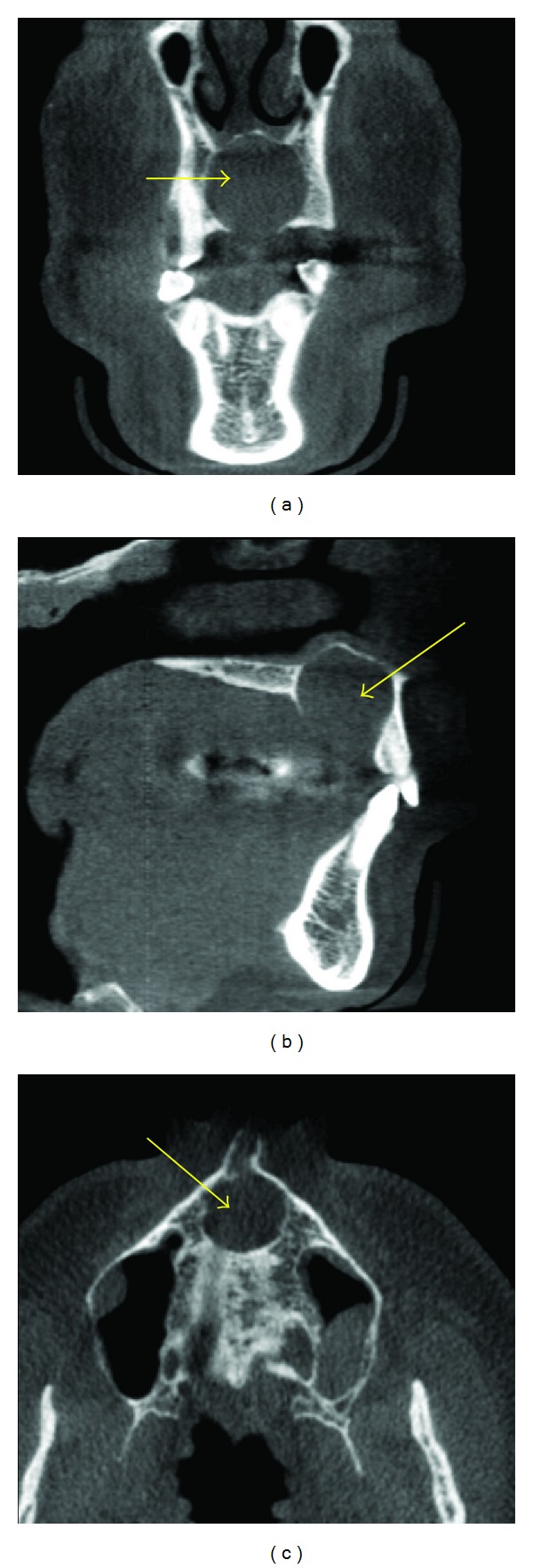
A nasopalatine canal cyst (arrows) is seen as a well-defined radiolucent entity in the maxilla in the midine in the regions of the nasopalatine canal. The inferior borders of this entity are not visualized.

The most common findings (783) were peridental in location. Periapical rarefying osteitis (281 subjects) was the most common finding followed by enostosis (136 subjects), graft material and/or sclerotic healing (108 subjects), retained root fragments both from deciduous and permanent teeth (100 subjects), impactions not including third molars (66 subjects), restorative material in the periapical regions of teeth (59 subjects), and external resorption (42 subjects). Relatively fewer common findings included sclerosing osteitis (17 subjects), oroantral fistula (14 subjects), hypercementosis (13 subjects), cemento-osseous dysplasia (Figure 3) (10 subjects), hyperplastic dental follicle (8 subjects), simple bone cyst (7 subjects), residual cyst (7 subjects), dentigerous cyst (Figure 4) (6 subjects), idiopathic osteosclerosis (5 subjects), cementoblastoma, (4 subjects), nasopalatine canal cyst (3 subjects) (Figure 5), reactive hyperplastic osteitis (2 subjects), keratocystic odontogenic tumor (2 subjects), osteomyelitis (2 subjects), and osteonecrosis both radiation induced (1 subject) and bisphosphonate induced (1 subjects). 

The paranasal sinuses were the location of the next common findings (605 findings). Mucositis/sinusitis/mucus retention pseudocysts (grouped as one category) were the most common findings (551 subjects). Surgical changes (29 subjects), hypoplastic sinuses (21 subjects) and osteoma in one of the paranasal sinuses (4 subjects) were the other findings in the paranasal sinuses.

Osteoarthrosis (240 subjects) was the most common condition found in the cervical vertebrae. Osseous screws (3 subjects), fusion of cervical vertebrae (1 subject) and non-segmentation of C2-3 vertebrae (1 subject) were the other findings visualized in the vertebrae.

Osteoarthrosis (158 subjects) was also the most common condition found in the TMJs and associated structures. Coronoid hyperplasia (17 subjects), Condylar hyperplasia (3 subjects) and condylar hypoplasia (2 subjects) accounted for the other findings in the TMJs region.

**Figure 6:**
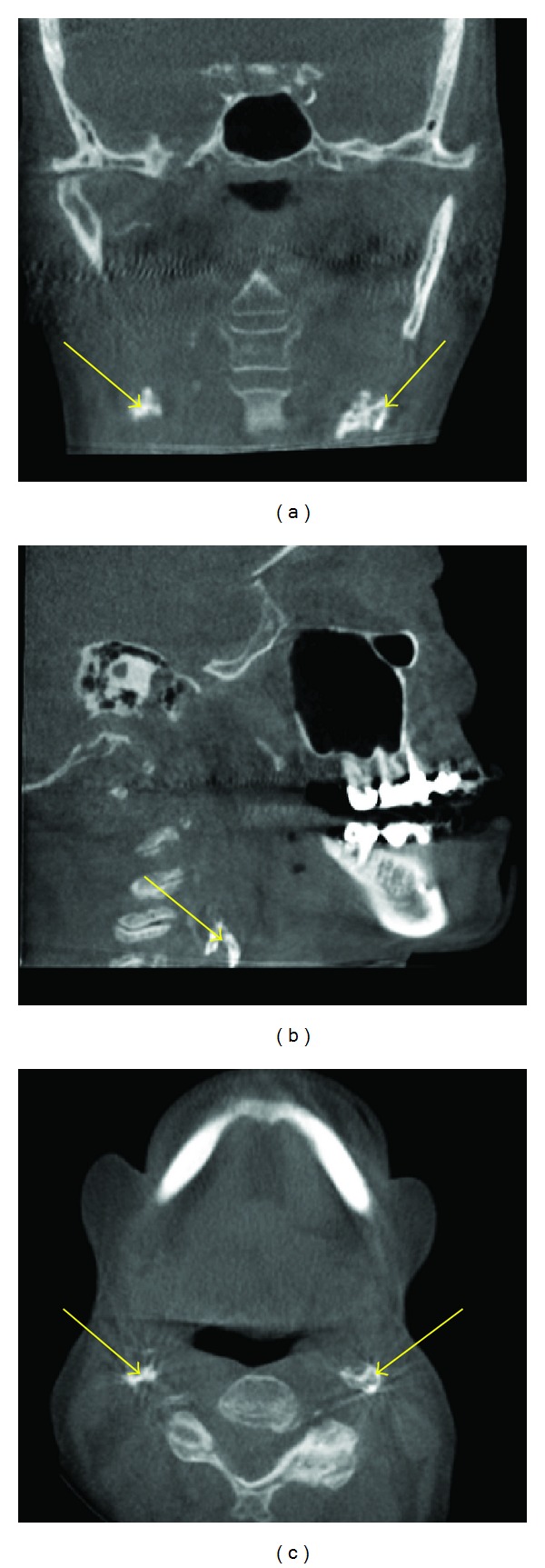
Carotid artery calcifications (arrows) seen as multiple small radiopaque linear entities taking the outline of the shape of a vessel in the cervical regions on these MPR images.

Pineal gland calcifications (147 subjects), tonsilliths (92) subjects were the most common findings amongst the different calcifications seen. Carotid artery calcifications (Figure 6) (cervical and/or intracranial calcifications—57 subjects) were the most significant amongst the different calcifications visualized in these scans. Males (28 of the 382 subjects) had a significantly higher percentage of carotid artery calcifications when compared to females (29 of the 618 subjects). Osteoma cutis (23 subjects), sialoliths (4 subjects) and vertebral artery calcification (1 subjects), and a variety of dystrophic calcifications including in the temporal regions (3 subjects), adenoids (2 subjects), and epiglottis regions (2 subjects), were the other calcifications encountered in these scans.

**Figure 7:**
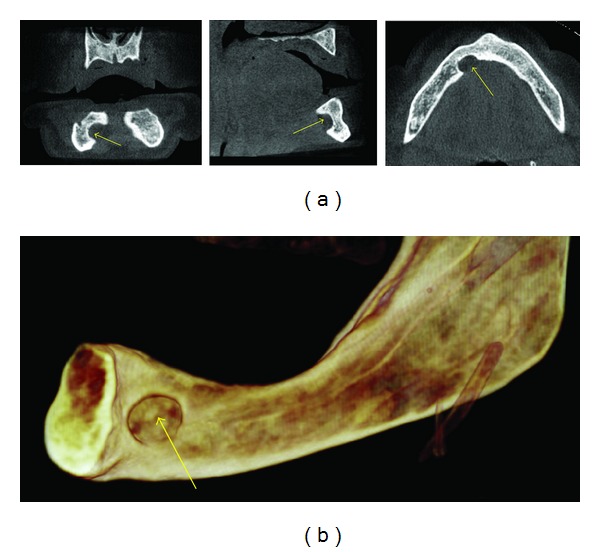
An anterior Stafne defect (ASD) seen as a well-defined area of scooped out bone in the region of the sublingual salivary gland on the right side of the lingual aspect of the mandible on these MPR (arrows) and 3D images.

**Figure 8:**
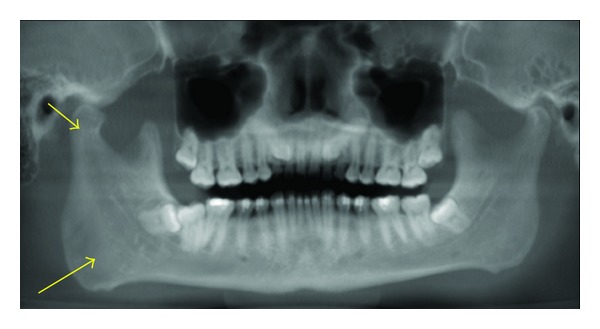
Hemihyperplasia seen as a much larger right side of the mandible on this panoramic reconstruction CBCT image.

There was a variety of other findings that were not grouped into any particular category in our study. Adenoidal hyperplasia (107 subjects) was the common other finding. There were also soft tissue swellings (9 subjects) in the airway regions other than in the adenoids. Large palatal tori (8 subjects), cleft palate (5 subjects), shot gun wound (4 subjects), hair artifacts (4 subjects), hearing aids (4 subjects), osteopenia (4 subjects), retained suture material in the jaws (4 subjects), nose rings (3 subjects), earrings (3 subjects), malignancy (3 subjects), Stafne defect (Figures 7(a) and 7(b)) (3 subjects), mandibular hemihyperplasia (Figure 8) (2 subjects), surgical drain in the soft tissue of the brain (1 subject), surgical staples in the neck (1 subject), nut notch (1 subject), and an implant impinging on the borders of the inferior alveolar canal (1 subject).

**Figure 9:**
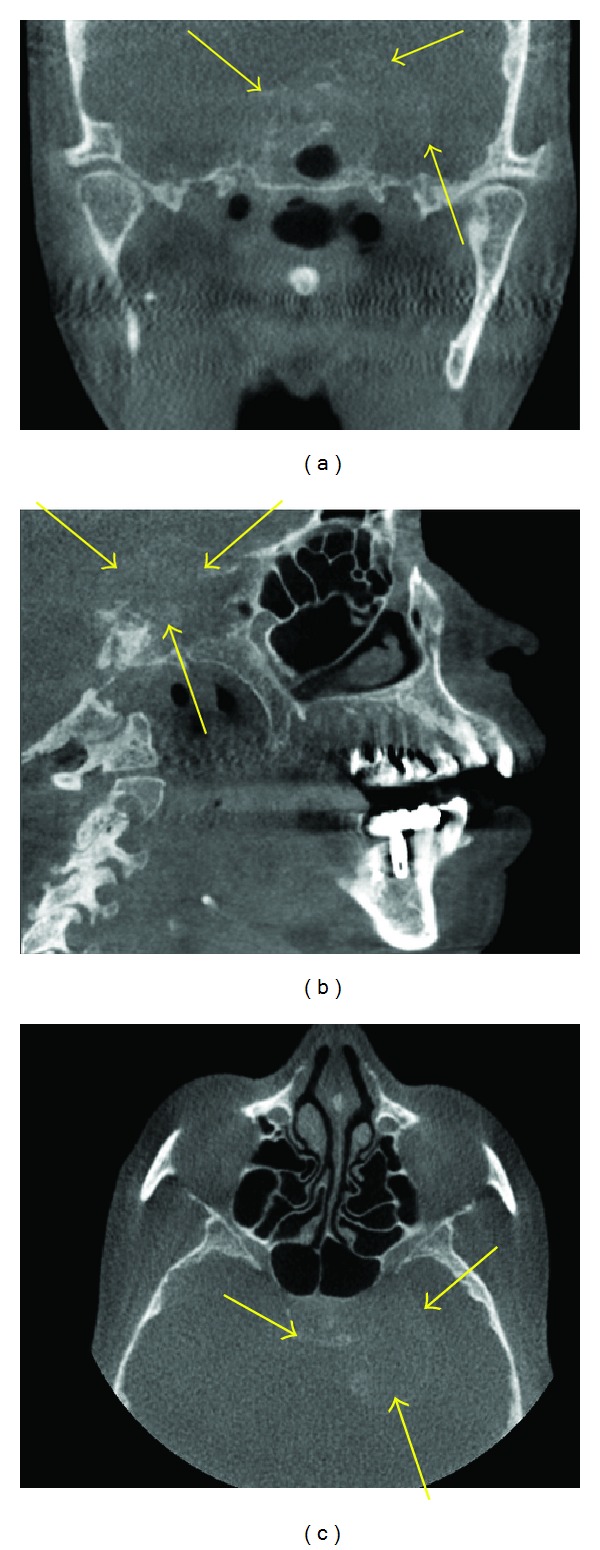
A malignancy seen as a soft tissue mass (arrows) in the sella region, which is causing extensive destruction in the osseous components of the bone in the sella region and encroaching into the adjacent areas on these MPR images.

**Figure 10:**
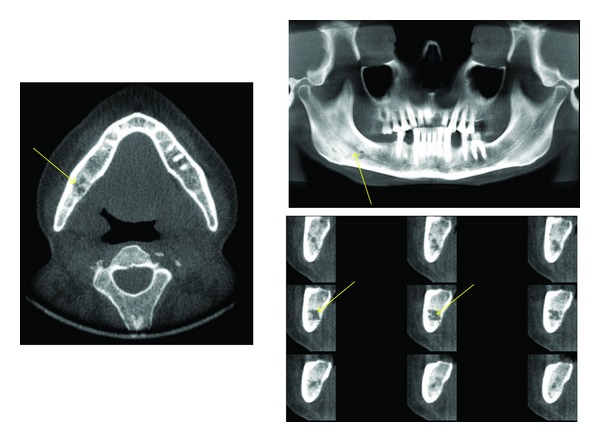
A metastatic lesion seen as multiple poorly defined radiolucent areas (arrows) in the right side of the mandible causing loss of the cortical borders of the right inferior alveolar canal on these axial, panoramic, and orthoradial images.

The most significant of all these findings were three malignancies (2 males and 1 female) found incidentally. One in the sella region had caused extensive destruction of the sella turcica (Figure 9), two subjects had metastatic lesions in the mandible, one from the prostate and one from breast (Figure 10). 

## 5. Discussion

Of the 1000 scans that were reviewed only 57 had no osseous pathosis or incidental findings. 943 scans showed findings in the primary regions of interest and/or outside the regions of interest and 77 different conditions were visualized in these scans both in and outside the areas of interest. Often the scans had incidental findings in more than one area. The percent of incidental findings in our study (94.3) was similar to that of the the other studies done by Cağlayan and Tozoğlu [4] (92.8%) and Price et al. [5] [90.7%] and greater than the study by Cha et al. (24.5%) [6].

One of the advantages of the current study when compared to other studies like Price et al. is that in those subjects in whom there were diseases that were required following biopsy, the findings in the original report were compared with findings from the histopathological evaluation and the prevalence of the different diseases was based on the histopathological confirmation of the radiologic interpretation. This step the authors believe was the most significant when compared to other studies that evaluated incidental findings and had mentioned that this was one of the short comings of their study (Price et al.). 

Our study is the largest study looking at incidental findings using 1000 subjects compared to the studies by Price et al. (300 cases), Cağlayan and Tozoğlu (207 cases), Cha et al. (500 cases), and Pette et al. [7] (318 cases). Such a large sample provides a better clarification of the importance of reviewing CBCT scans thoroughly as significant diseases such as malignancies and also those diseases that are relatively rare are more likely to be included in the sample size, for example, the malignancy cases which were the most significant and immediately life threatening to the subjects. Our study also compares well with current published manuscripts by Pliska et al. [8] and Rogers et al. [9] and Pazera et al. [10] which all looked at incidental findings on CBCTs made on orthodontic patients. The advantage of our study in comparison to the above studies is that there is a greater likelihood of finding diseases when a wider age group of patients was included.

Some of the drawbacks of our study were that a single oral and maxillofacial radiologist reviewed all the images predominantly, although others were available for consultation. One of the other drawbacks of our study was that it included almost one half times (382 males to 618 females) the number of female subjects when compared to the males. It makes comparison of prevalence of various diseases somewhat more difficult than if there were equal number of subjects. 

## 6. Conclusions

From the wide and comprehensive scope of findings found inside and outside the primary areas of interest in the 1000 subjects, it can be concluded that it is essential that a person trained in advanced interpretation techniques in radiology interprets CBCT scans. It can also be concluded that these CBCT images need to be reviewed comprehensively.

## Figures and Tables

**Table 1:** Age and Sex distribution of subjects.

Age group	Number of subjects		
	Males	Females	Total
5 years and 11 months–10	12	13	25
11–20	78	101	179
21–30	21	30	51
31–40	29	62	91
41–50	53	94	147
51–60	86	134	220
61–70	64	133	197
71–80	35	44	79
81–87	4	7	11

Total	382	618	1000

**Table 2:** Table showing why CBCT scans were made on the subjects.

Reason for Scan	No of Subjects
Implants/Bone evaluation for implants	678
Impaction localization	110
Orthodontic records	67
Other possible pathosis	46
Evaluation of graft in the sinuses area/bone assessment for implants	40
Supernumerary teeth localization	31
Sinus evaluation prior to implants	17
TMJ assessment	11

Total	1000

**Table 3:** Summary of all the findings seen in the 1000 scans (318 males and 682 females).

Findings Visualized	Male	Female	Total
Variations in size, shape, and number of teeth			
Oligodontia	16	22	38
Supernumerary teeth	19	12	31
Transposition	2	3	5
Compound Odontoma	0	4	4
Microdontia	3	1	4
Dentin dysplasia	1	1	2
Dentinogenesis imperfecta with osteogenesis imperfecta	0	1	1
Amelogenesis imperfecta	1	0	1
Taurodontism	1	0	1
Gemination	1	0	1
Macrodontia	1	0	1

Periapical/parapical/peridental findings			
Rarefying osteitis	114	167	281
Enostosis	54	82	136
Graft material and sclerotic healing	35	73	108
Root fragments	45	55	100
Impactions (not including third molars)	29	37	66
Restorative material in the periapical regions of teeth	22	37	59
External resorption	22	20	42
Sclerosing osteitis	6	11	17
Oroantral fistulas	6	8	14
Hypercementosis	5	8	13
Cemento-osseous dysplasia	1	9	10
Hyperplastic dental follicle	3	5	8
Fibrous dysplasia	4	4	8
Simple bone cyst	3	4	7
Residual cyst	3	4	7
Dentigerous cyst	4	2	6
Osteosclerosis	2	3	5
Cementoblastoma	2	2	4
Nasopalatine canal cyst	3	0	3
Reactive hyperplastic osteitis	0	2	2
Keratocystic odontogenic tumor	1	1	2
Giant cell lesion	1	0	1
Osteomyelitis	1	1	2
Radioosteonecrosis	0	1	1
Chemoosteonecrosis	0	1	1

Pathosis/anatomical variants in the paranasal sinuses			
Mucositis/sinusitis/mucous retention pseudocysts	246	305	551
Surgical changes in the sinuses	13	16	29
Hypoplastic sinuses	8	13	21
Osteoma	1	3	4

Findings in cervical vertebrae region			
Osteoarthrosis	90	150	240
Osseous screws in vertebrae	1	2	3
Fusion of C2-3 cervical vertebrae	1	0	1
Nonsegmentation of C2-3 vertebrae	1	0	1

Findings in the TMJs region/associated structures			
Osteoarthrosis	42	116	158
Coronoid hyperplasia	8	9	17
Condylar hyperplasia	1	2	3
Condylar hypoplasia	2	0	2

Calcifications visualized in the volume			
Pineal gland calcifications	43	104	147
Tonsilliths	53	39	92
Carotid artery calcifications	28	29	57
Osteoma cutis	9	14	23
Sialoliths	1	3	4
Vertebral artery calcification	0	1	1

Other dystrophic calcifications			
Temporal regions	1	2	3
Adenoids	1	1	2
Epiglottis	1	1	2

Other findings			
Adenoidal hyperplasia	44	63	107
Soft tissue swellings in the airway region	2	7	9
Palatal tori	1	7	8
Cleft palate	4	1	5
Shot gun wound	0	4	4
Hair artifacts	0	4	4
Hearing aids	1	3	4
Osteopenia	1	3	4
Retained suture material in the jaws	2	2	4
Nose ring	0	3	3
Earrings	0	3	3
Malignancy	2	1	3
Stafne defect	2	1	3
Mandibular hemihyperplasia	2	0	2
Unhealed fracture	1	0	1
Surgical drain in the soft tissue of brain	1	0	1
Surgical staples in the neck	1	0	1
Nut notch	1	0	1
Implant impinging on borders of the inferior alveolar canal	1	0	1
